# Supplementary material for: Preparation of oxygen-sensitive proteins for high-resolution cryoEM structure determination using blot-free vitrification
Source: Nat Commun. 2025 Apr 14;16:3528. doi: 10.1038/s41467-025-58243-1 (PMC11997128; doi:10.1038/s41467-025-58243-1)
Supplement: Supplementary file 1 — Supplementary Information [file 41467_2025_58243_MOESM1_ESM.pdf]

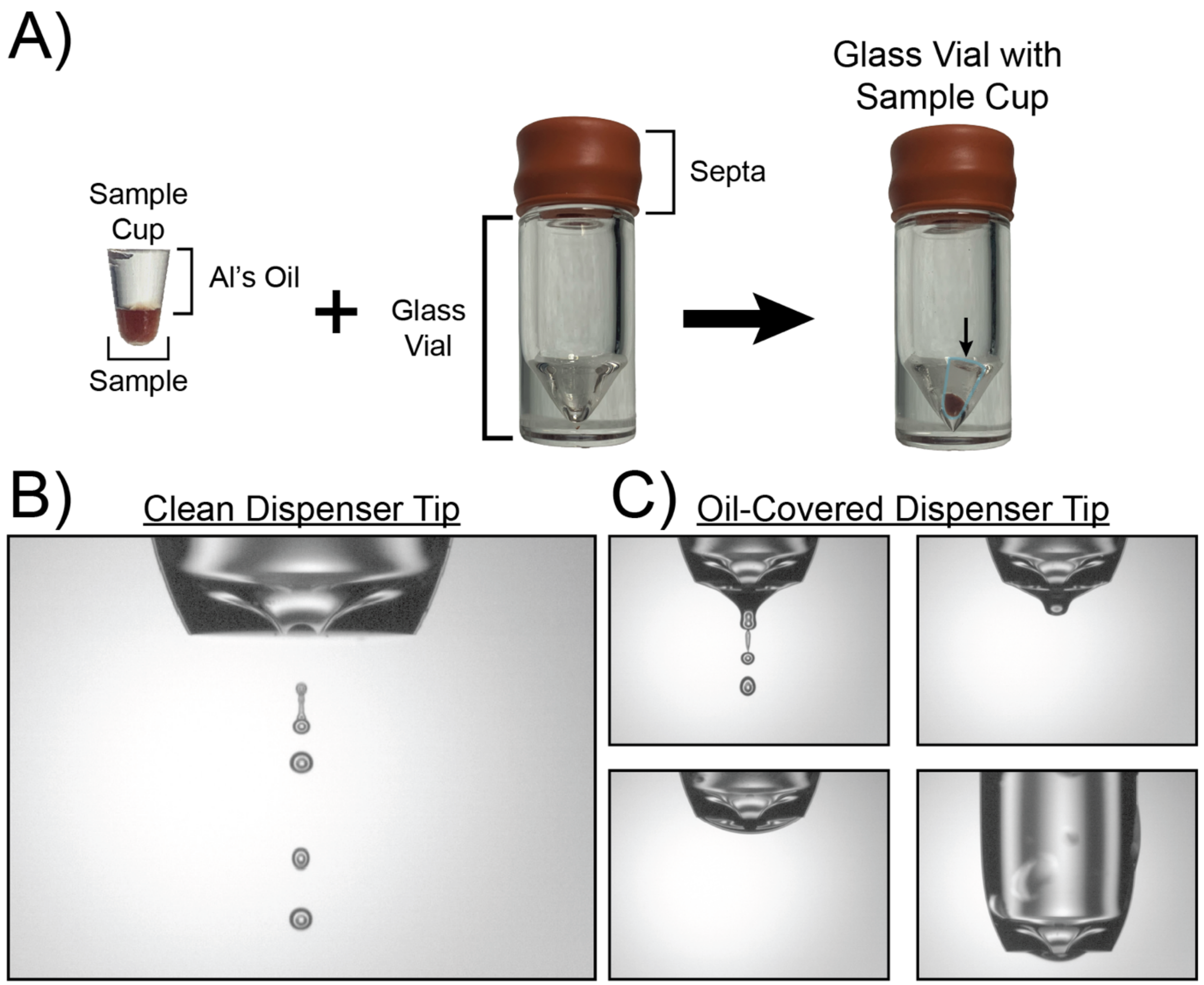

Figure S1: Images of critical steps in the (an)aerobic workflow. A) Images of a Hb sample in a chameleon sample cup with the protective Al's oil layer on top. The chameleon sample cup is prepared within an anaerobic environment and transferred into gas-tight glass vial and sealed with a rubber septum. B-C) Images from the chameleon software displaying either a clean functioning dispenser tip (B) or a tip with residual oil on the outside (C) The residual oil can be removed using dispenser washing or wiping steps to yield a clean dispenser tip (B).

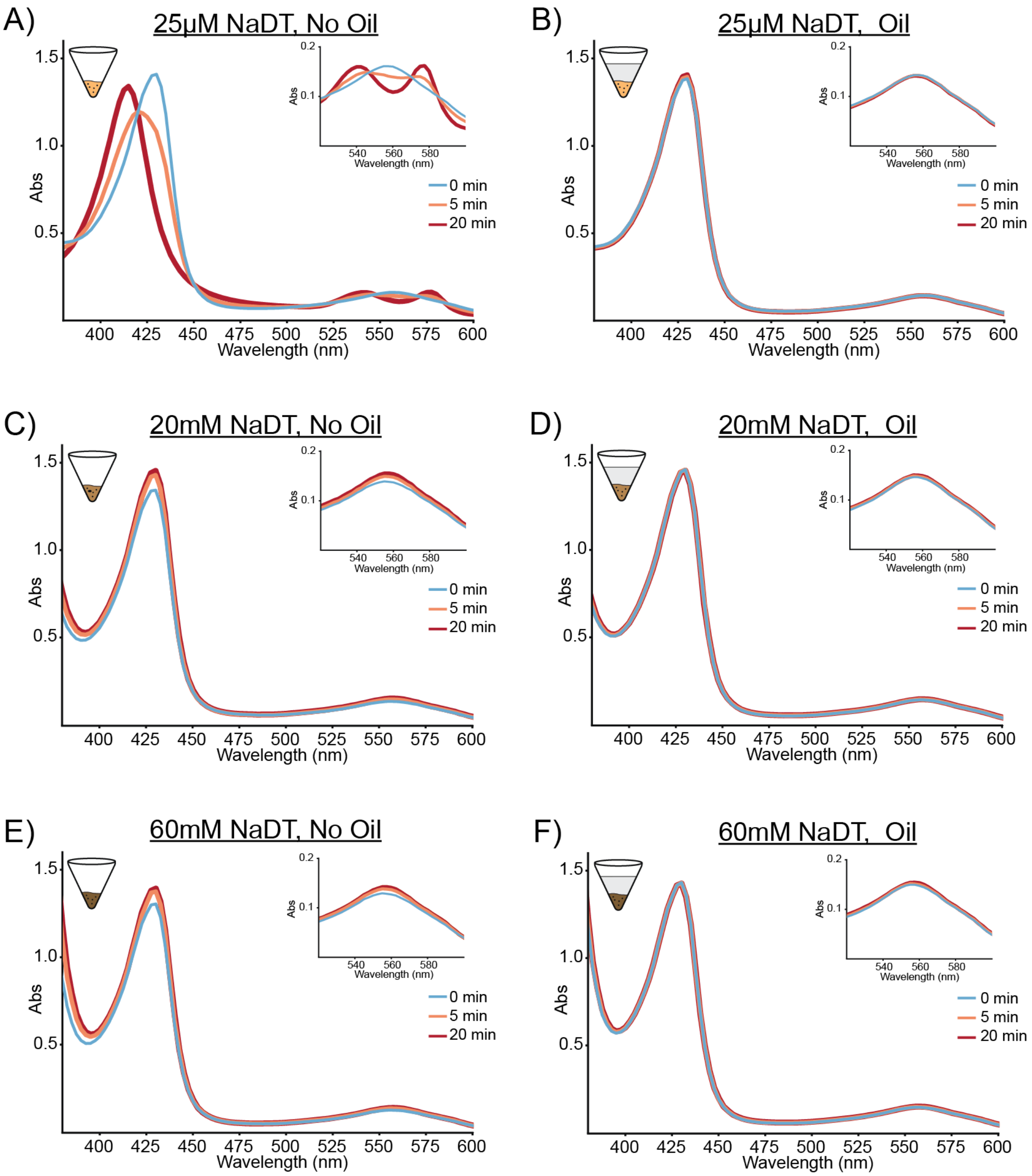

Figure S2: UV-Vis-based oxygen perfusion assay using human deoxyHb under varying conditions. For each sample, the anaerobic cuvette containing deoxyHb was opened to air and UV-Vis spectra were recorded from 350-600 nm over the course of 20 minutes. A,B) Timecourse of deoxyHb with 25 μM NaDT in sample buffer without and with a protective oil layer, respectively. C,D) Timecourse of deoxyHb with 20 mM NaDT in sample buffer without and with a protective oil layer, respectively. E,F) Timecourse of deoxyHb with 25 μM NaDT in sample buffer without and with a protective oil layer, respectively. For each assay, an inset of the 525-550 nm region is shown. For each assay, UV-Vis spectra at time 0 min (blue), 5 min (orange), and 20 min (red) are shown.

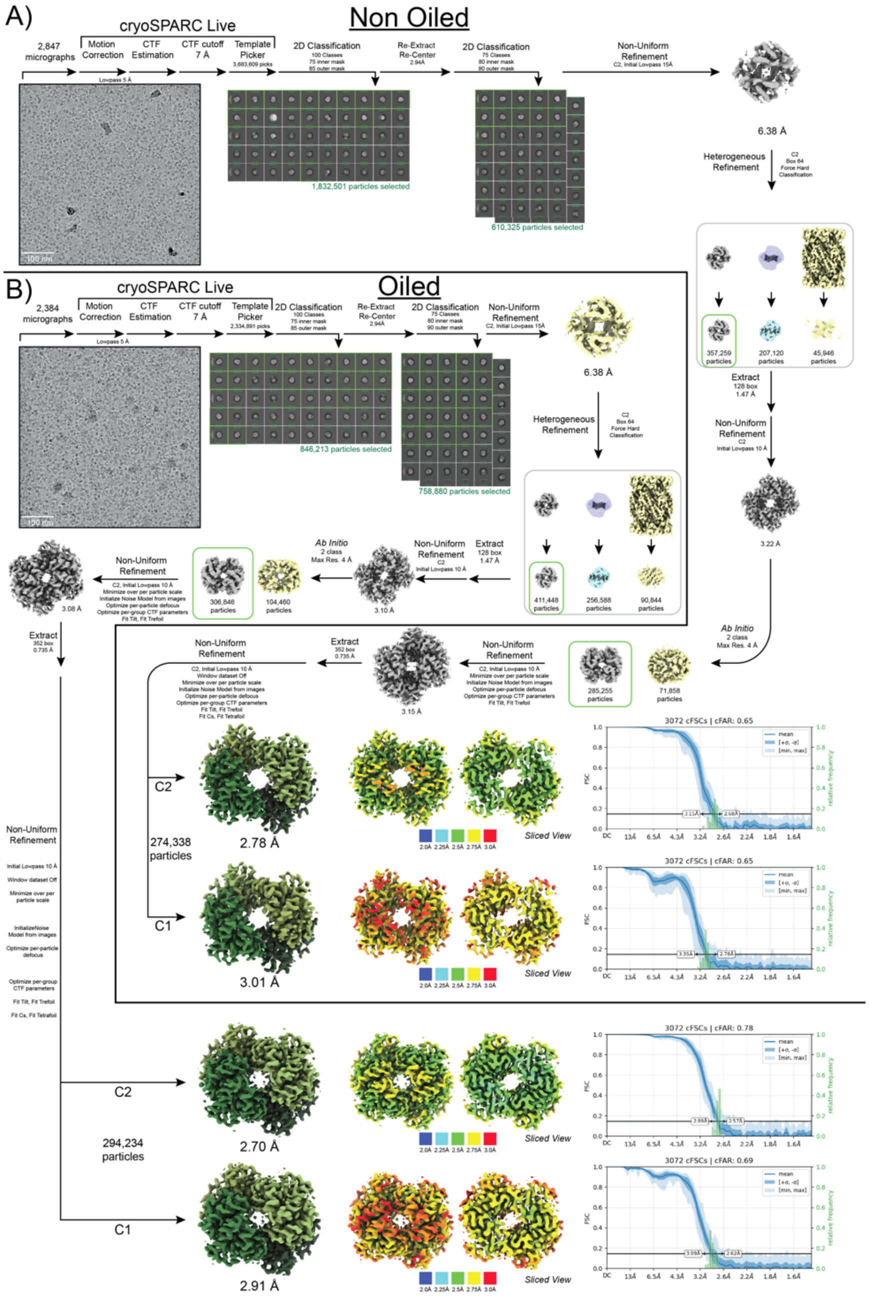

Figure S3: CryoEM data processing workflow for non-oiled and oiled metHb structures. A) Single-particle cryoEM data processing workflow for the 2.78 Å resolution non-oiled metHb structure. B) Single-particle cryoEM data processing workflow for the 2.71 Å resolution oiled metHb structure. 2,847 micrographs and 2,384 micrographs of non-oiled and oiled metHb, respectively, were collected and processed using similar strategies. Briefly, particle coordinates were obtained via template picking and extracted downsampled 4x prior to successive rounds of 2-D classification. Particles within the best classes were 3-D refined followed by a heterogeneous refinement using two Hb volumes and the 20S proteasome from *T. thermophilus* (EMDB-4877). Particles in the best class were carried downstream for 3-D refinement without downsampling with per-particle CTF and aberration refinements. For the final refinement, C1 and C2 symmetry were employed. Both non-oiled and oiled structures comprised similar final particle numbers, 275K versus 295K, respectively, and refined to similar FSC-estimated resolution with similar viewing distributions as estimated by 3-D FSC. Each of the final structures are colored by local resolution and the 3-D FSC plots are shown.

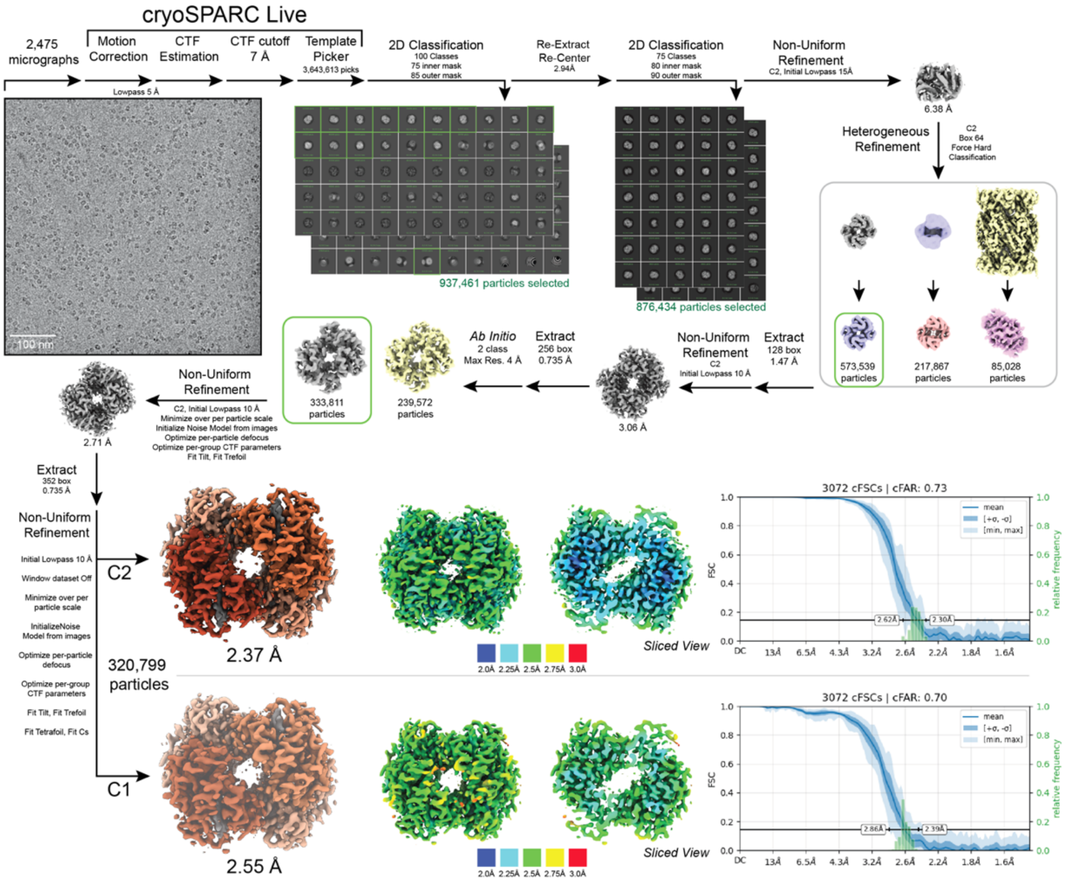

Figure S4: CryoEM data processing workflow for the very low NaDT (25 μM), oxyHb structure. Single-particle cryoEM data processing workflow for the 2.37 Å resolution oxyHb structure that was obtained in 25 μM NaDT under oil. 2,475 micrographs were collected and processed using a similar strategy as the metHb structures in Fig S3. Briefly, particle coordinates were obtained via template picking and extracted downsampled 4x prior to successive rounds of 2-D classification. Particles within the best classes were 3-D refined followed by a heterogeneous refinement using two Hb volumes and the 20S proteasome from *T. thermophilus* (EMDB-4877). Particles in the best class were carried downstream for 3-D refinement without downsampling with per-particle CTF and aberration refinements. For the final refinement, C1 and C2 symmetry were employed. Each of the final structures are colored by local resolution and the 3-D FSC plots are shown.

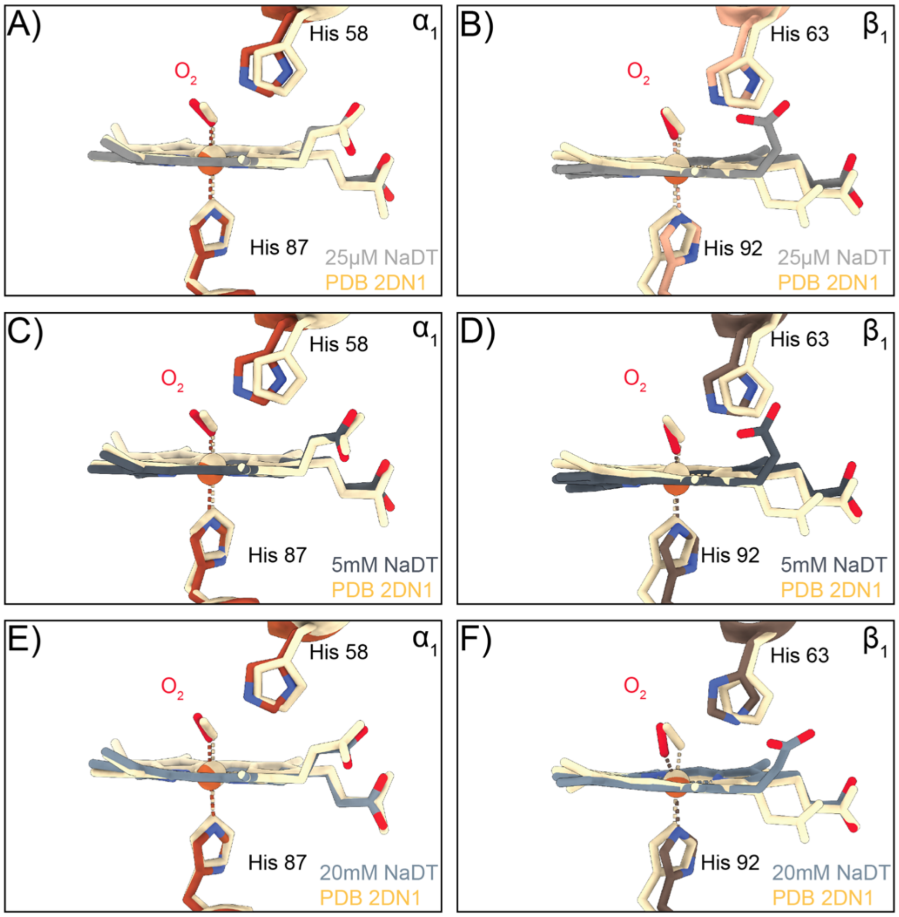

Figure S5: Comparison of oxygen binding geometry in our oxyHb and partially-oxygenated Hb cryoEM structures vs oxyHb determined using X-ray crystallography structure (PDB: 2DN1). A-B) The atomic models for the hemes of the α_1 subunit (A) and β_1 subunit (B) (colored gray) and bound oxygen (colored red) overlapped with the heme and bound oxygen from the X-ray crystallography structure (PDB: 2DN1), shown in wheat detailing similar geometries C-D) The atomic models for the hemes of the α_1 subunit (C) and β_1 subunit (D) (colored blue-gray) and bound oxygen (colored red) overlapped with the heme and bound oxygen from the X-ray crystallography structure (PDB: 2DN1), shown in wheat detailing similar geometries. The atomic models for α_1H58, α_1H87, $\beta_1$63, and $\beta_1$92 are also shown. E-F) The atomic models for the hemes of the α_1 subunit (E) and β_1 subunit (F) (colored gray) and bound oxygen (colored wheat) overlapped with the heme and bound oxygen from the X-ray crystallography structure (PDB: 2DN1), shown in wheat detailing similar geometries. The atomic models for α_1H58, α_1H87, $\beta_1$63, and $\beta_1$92 are also shown.

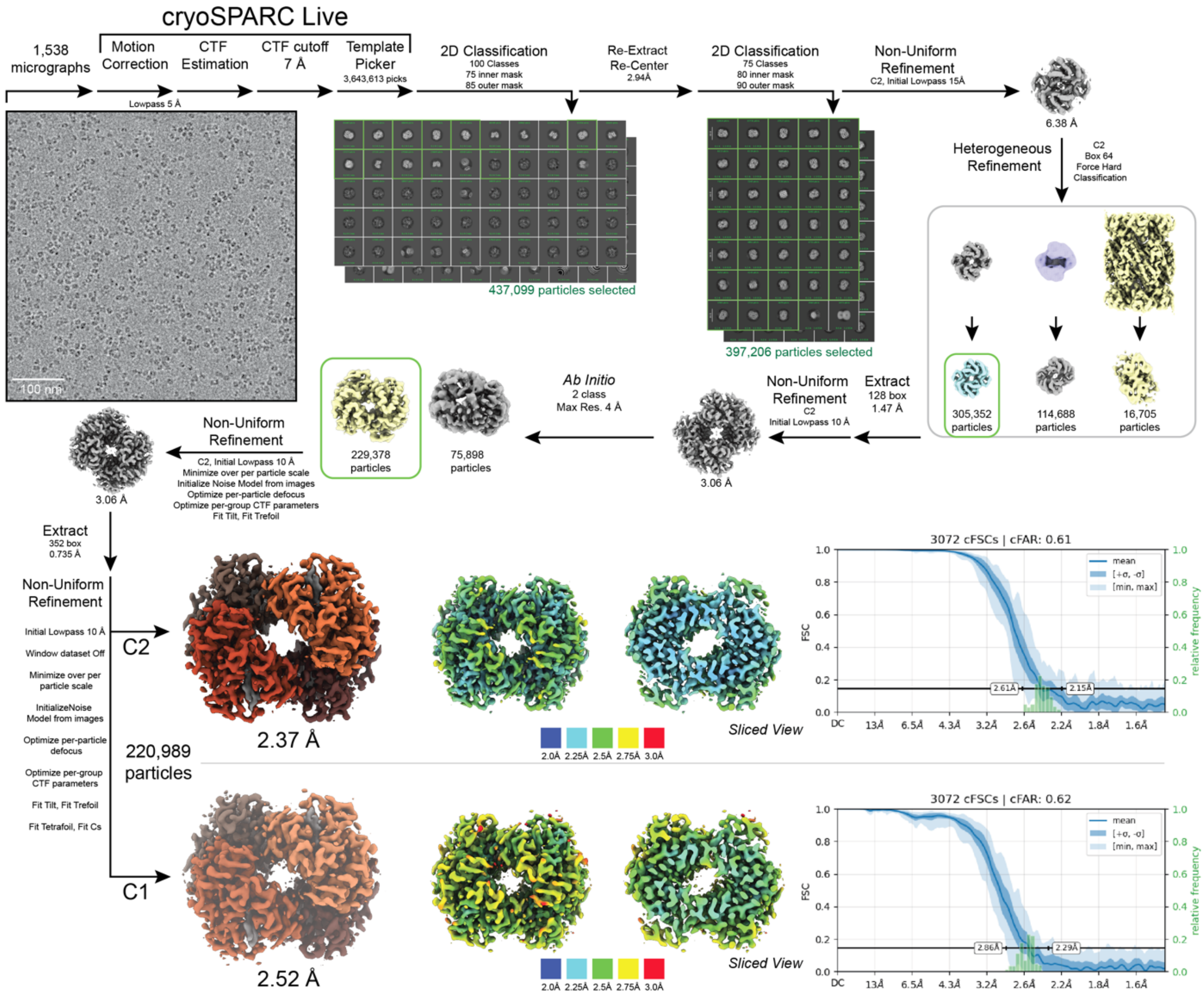

Figure S6: CryoEM data processing workflow for the low NaDT (5 mM), oxyHb structure. Single-particle cryoEM data processing workflow for the 2.37 Å resolution oxyHb structure that was obtained in 5 mM NaDT under oil. 1,538 micrographs were collected and processed using a similar strategy as the very low NaDT Hb structures in Fig S4. Briefly, particle coordinates were obtained via template picking and extracted downsampled 4x prior to successive rounds of 2-D classification. Particles within the best classes were 3-D refined followed by a heterogeneous refinement using two Hb volumes and the 20S proteasome from *T. thermophilus* (EMDB-4877). Particles in the best class were carried downstream for 3-D refinement without downsampling with per-particle CTF and aberration refinements. For the final refinement, C1 and C2 symmetry were employed. Each of the final structures are colored by local resolution and the 3-D FSC plots are shown.

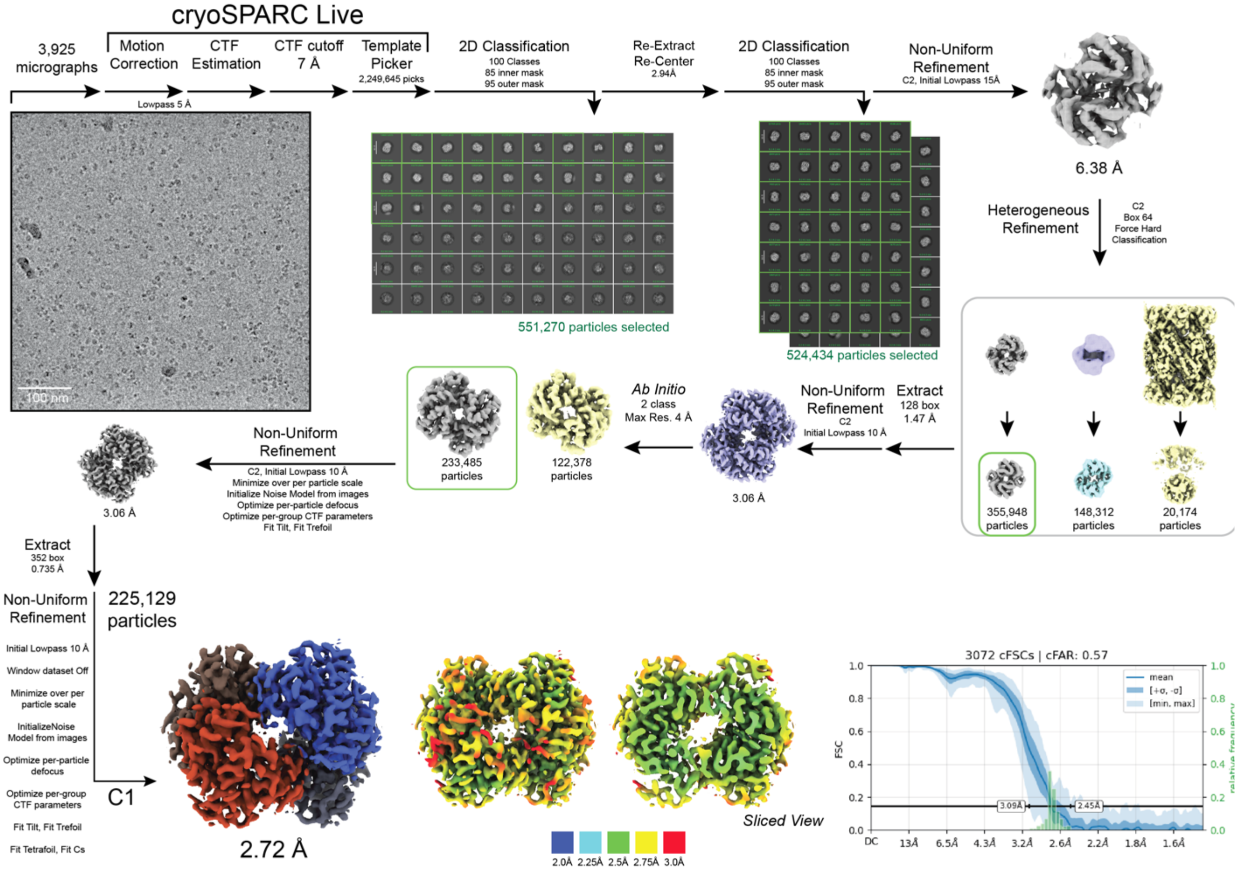

Figure S7: CryoEM data processing workflow for the medium NaDT (20 mM), partially-oxygenated Hb structure. Single-particle cryoEM data processing workflow for the 2.72 Å resolution partially-oxygenated (mixed) Hb structure that was obtained in 20 mM NaDT under oil. 3,925 micrographs were collected and processed using a similar strategy as the metHb structures in Fig S3. Briefly, particle coordinates were obtained via template picking and extracted downsampled 4x prior to successive rounds of 2-D classification. Particles within the best classes were 3-D refined followed by a heterogeneous refinement using two Hb volumes and the 20S proteasome from *T. thermophilus* (EMDB-4877). Particles in the best class were carried downstream for 3-D refinement without downsampling with per-particle CTF and aberration refinements. For the final refinement, C1 symmetry was employed. The final structure is colored by local resolution and the 3-D FSC plot is shown.

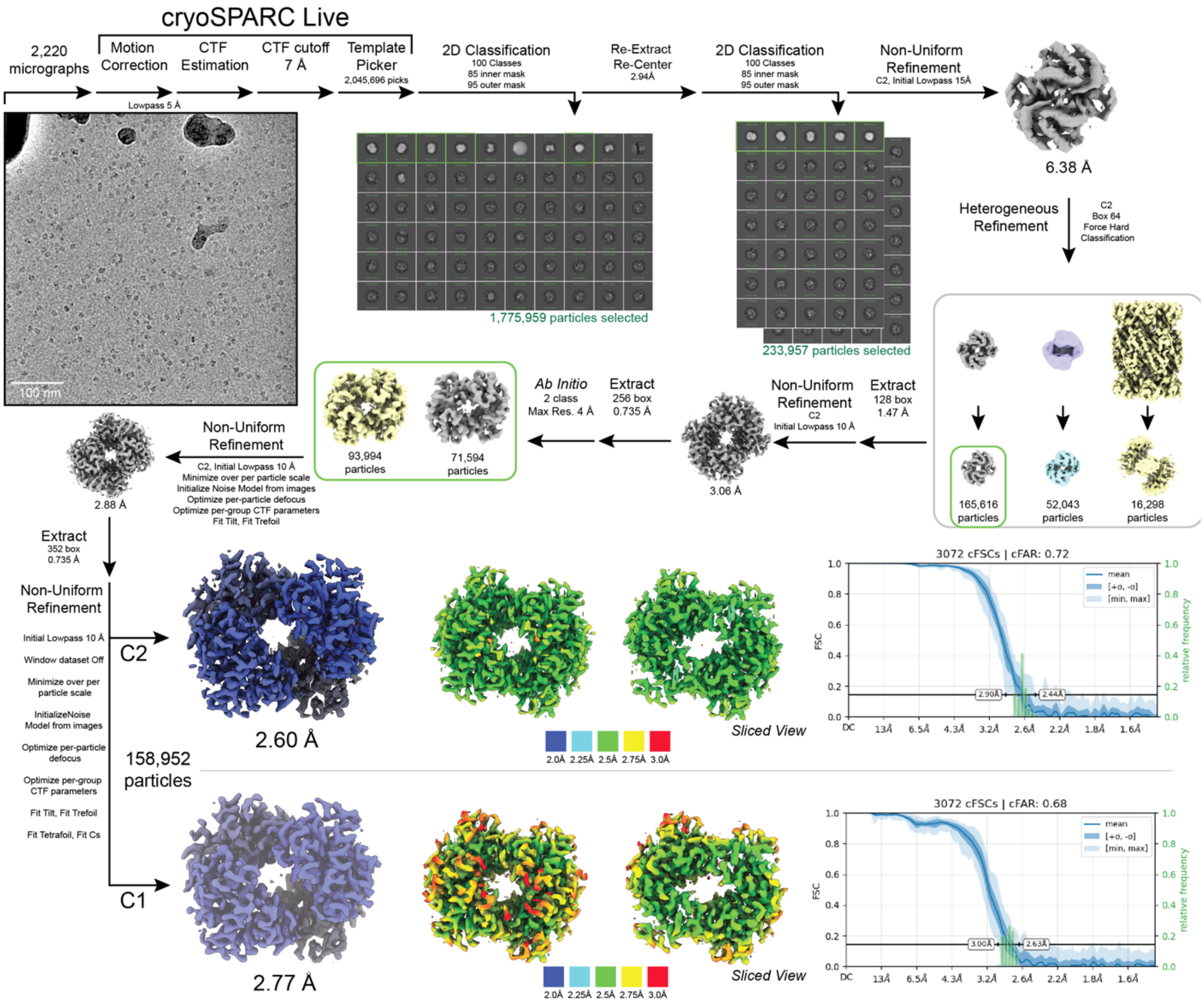

Figure S8: CryoEM data processing workflow for the high NaDT (60 mM), deoxyHb structure. Single-particle cryoEM data processing workflow for the 2.60 Å resolution deoxyHb structure that was obtained in 60 mM NaDT under oil. 2,220 micrographs were collected and processed using a similar strategy as the metHb structures in Fig S3. Briefly, particle coordinates were obtained via template picking and extracted downsampled 4x prior to successive rounds of 2-D classification. Particles within the best classes were 3-D refined followed by a heterogeneous refinement using two Hb volumes and the 20S proteasome from *T. thermophilus* (EMDB-4877). Particles in the best class were carried downstream for 3-D refinement without downsampling with per-particle CTF and aberration refinements. For the final refinements, C1 and C2 symmetry were employed. Each of the final structures are colored by local resolution and the 3-D FSC plots are shown.

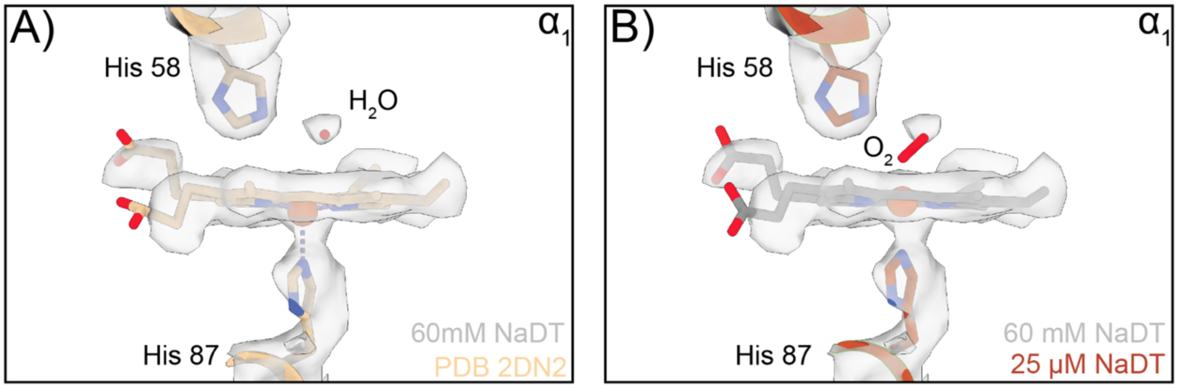

Figure S9: Water density in the distal pocket of the deoxyHb structure. A) CryoEM density of the 60 mM NaDT deoxyHb structure with the 1.25 Å deoxyHb X-ray atomic model (PDB: 2DN2) docked in place showing a well-defined water molecule above the ferrous unliganded heme. B) Same cryoEM density from (A) with our oxyHb atomic model docked in showing the poor fit of the oxygen ligand.

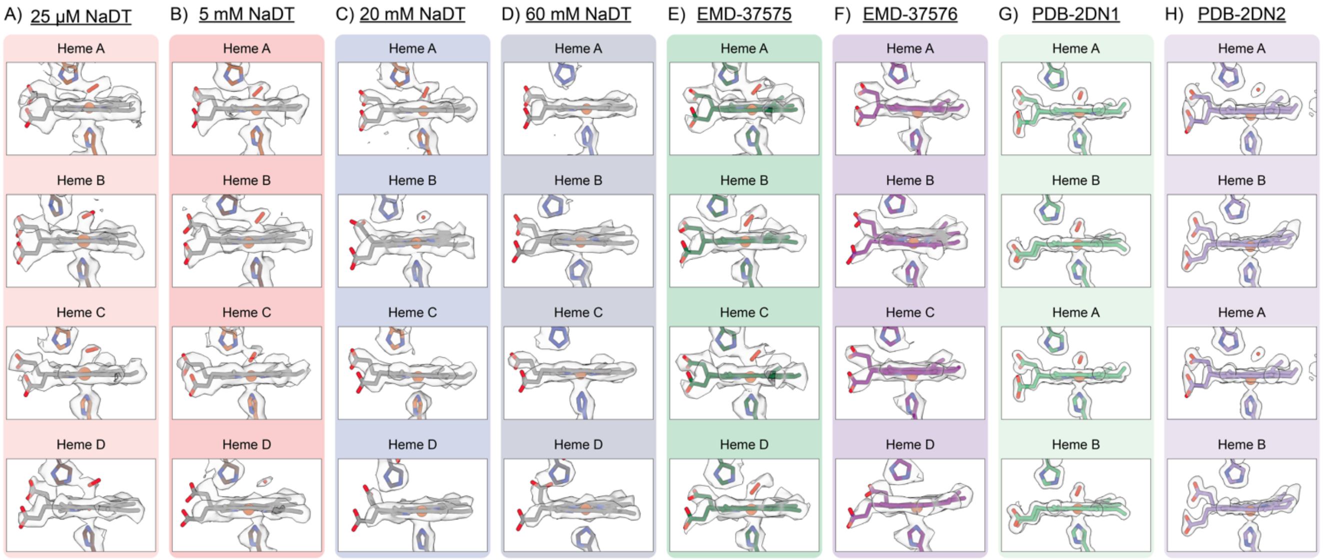

Figure S10: Comparison of Heme Densities. A-D) EM densities and corresponding atomic models for each Hb heme determined in this study are shown. E-F) The EM densities and atomic models of oxy human Hb (E) and deoxy human Hb (F) from Takahashi *et al*. 2024 are shown. G-H) X-ray crystallography electron density ($2F_o$-F_c) of oxy and deoxy Hb from Park *et al.,* 2006.

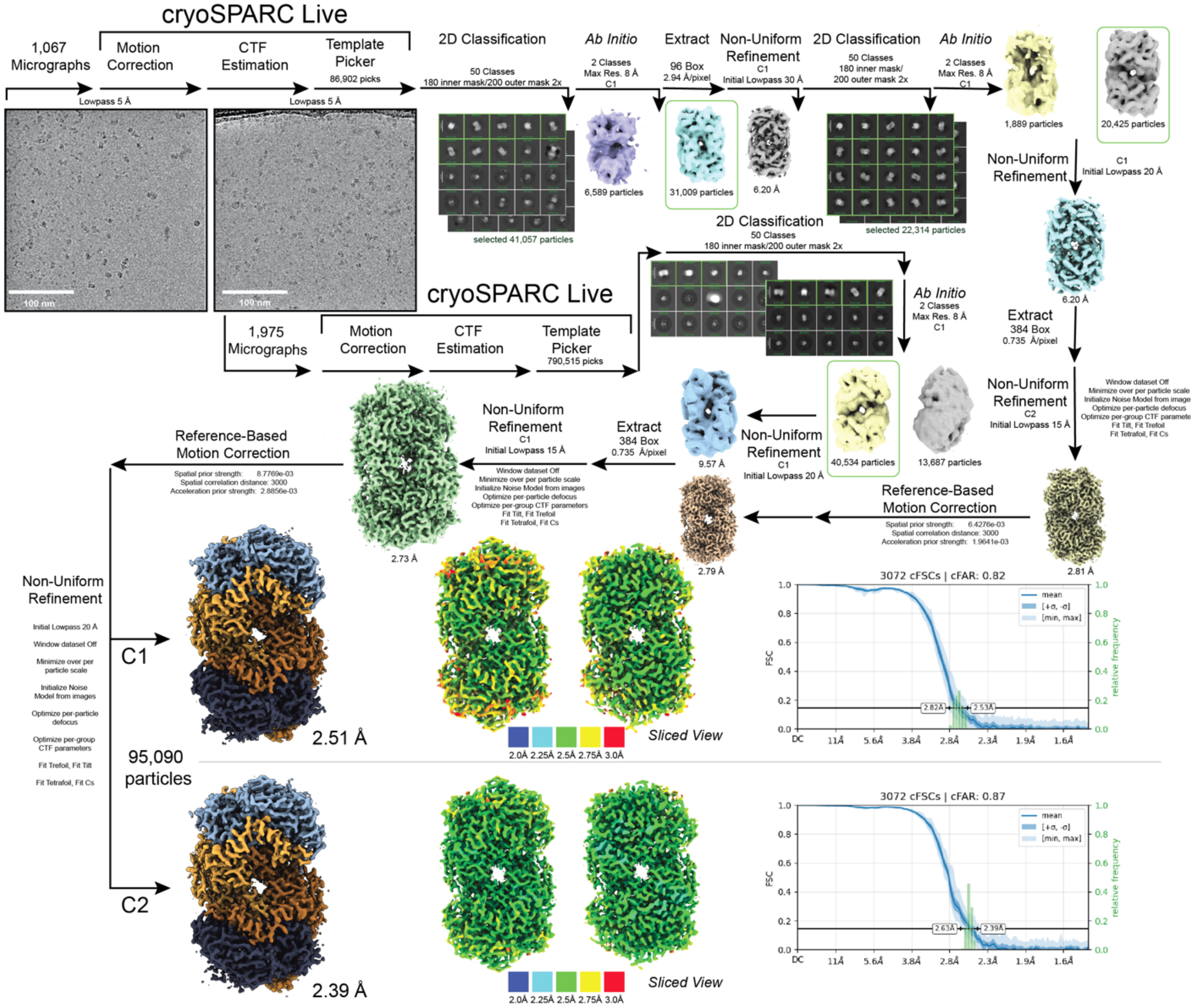

Figure S11: CryoEM data processing workflow for the oxidized P^{2+} *Av*MoFeP structure. Single-particle cryoEM data processing workflow for the 2.39 Å resolution oxidized P^{2+} *Av*MoFeP structure that was obtained in without the protective oil layer and without NaDT in the cryoEM buffer. 1,067 and 1,975 micrographs were collected across two collections and processed using a similar strategies before being combined at the end. Briefly, particle coordinates were obtained via template picking and extracted downsampled 4x prior to successive rounds of 2-D classification. Particles within the best classes were subjected to a 2-class *ab initio* model generation. Particles in the best class were then subjected to another round of 2-D classification and 2-class *ab initio* prior to 3-D refinement of the best particles without downsampling. Reference-based motion correction was performed for each particle set before combining for a non-uniform refinement with per-particle CTF and aberration refinements. For the final refinements, C1 and C2 symmetry were employed. Each of the final structures are colored by local resolution and the 3-D FSC plots are shown.

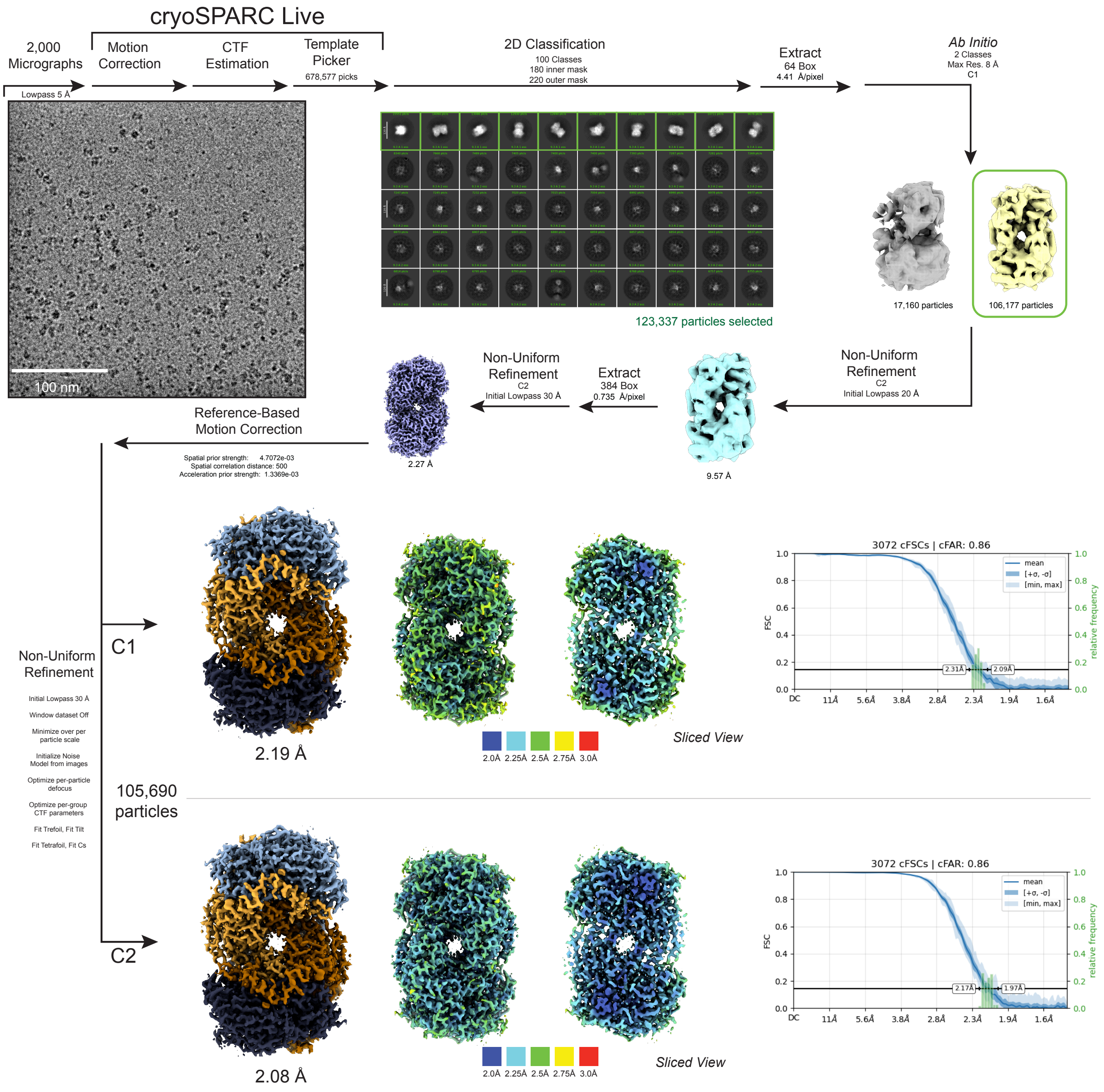

Figure S12: CryoEM data processing workflow for the reduced P^N *Av*MoFeP structure in the presence of 60 mM NaDT. Single-particle cryoEM data processing workflow for the 2.08 Å resolution reduced P^N *Av*MoFeP structure that was obtained with the protective oil layer and 60 mM NaDT in the cryoEM buffer. 2,000 micrographs were collected processed using a similar strategy as the oxidized P^{2+} MoFeP structure. For the final refinements, C1 and C2 symmetry were employed. Each of the final structures are colored by local resolution and the 3-D FSC plots are shown.

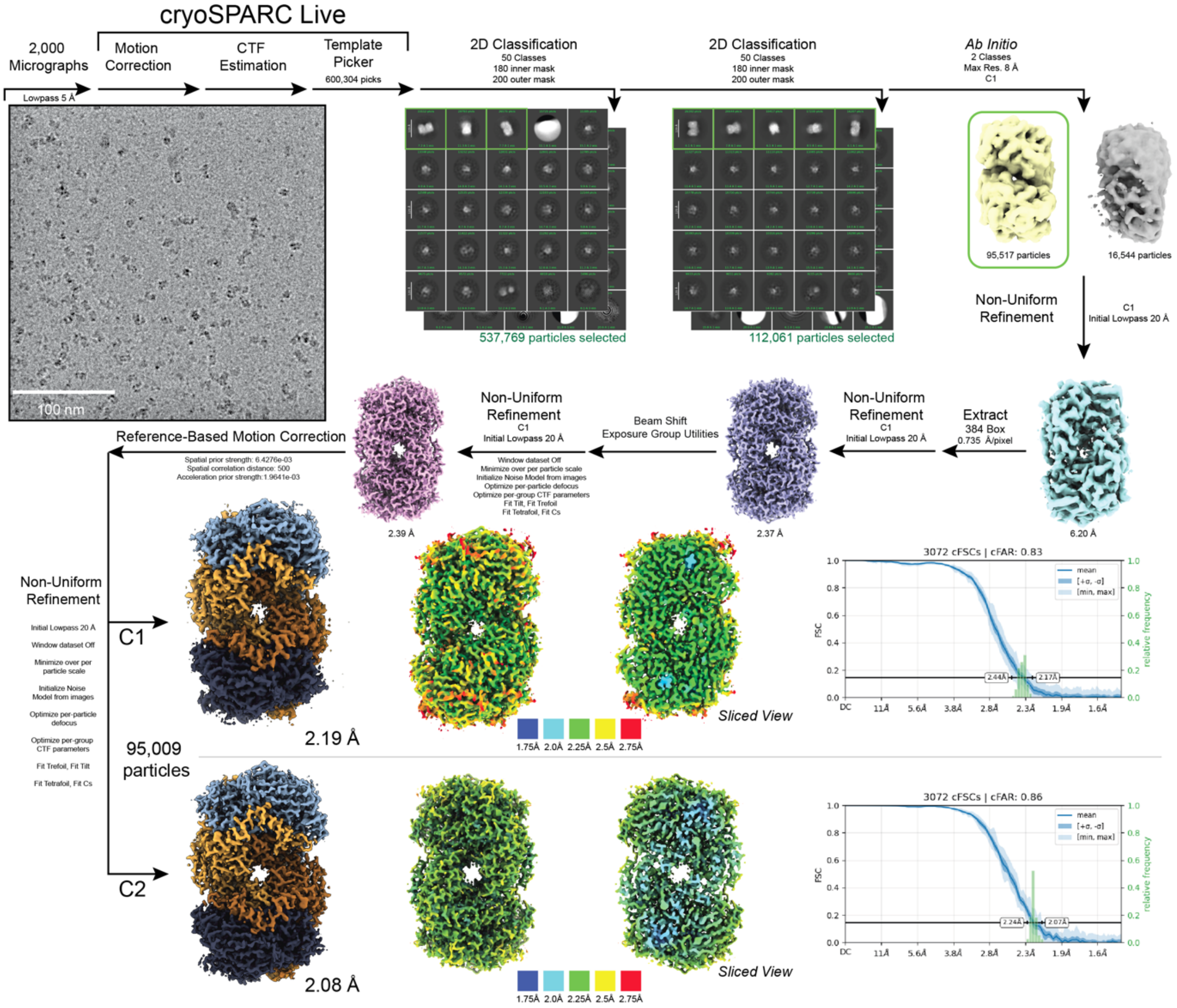

Figure S13: CryoEM data processing workflow for the reduced P^N *Av*MoFeP structure in the presence of 20 mM NaDT.. Single-particle cryoEM data processing workflow for the 2.19 Å resolution reduced P^N *Av*MoFeP structure that was obtained with the protective oil layer and 20 mM NaDT in the cryoEM buffer. 2,000 micrographs were collected processed using a similar strategy as the oxidized P^{2+} MoFeP structure. For the final refinements, C1 and C2 symmetry were employed. Each of the final structures are colored by local resolution and the 3-D FSC plots are shown.

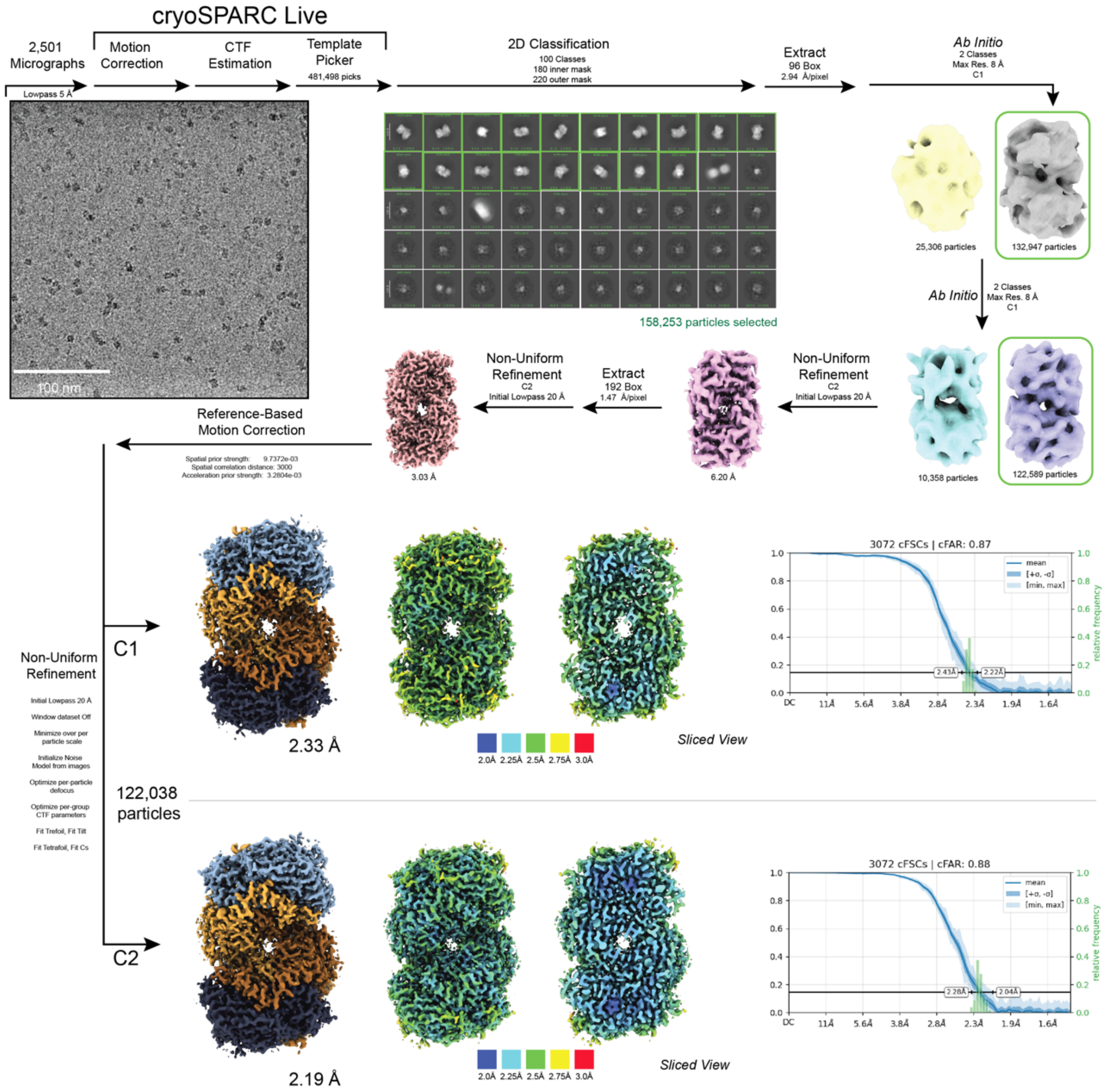

Figure S14: CryoEM data processing workflow for the reduced P^N *Av*MoFeP structure in the presence of 5 mM NaDT. Single-particle cryoEM data processing workflow for the 2.19 Å resolution reduced P^N *Av*MoFeP structure that was obtained with the protective oil layer and 5 mM NaDT in the cryoEM buffer. 2,501 micrographs were collected processed using a similar strategy as the oxidized P^{2+} MoFeP structure. For the final refinements, C1 and C2 symmetry were employed. Each of the final structures are colored by local resolution and the 3-D FSC plots are shown.

	No Oil		Under Oil		Very Low (25 µM) NaDT		Low (5 mM) NaDT		Medium (20 mM) NaDT		High (60 mM) NaDT	
**Data Collection**												
Magnification	165 kx		165 kx		165 kx		165 kx		165 kx		165 kx	
Voltage (kV)	300		300		300		300		300		300	
Spherical Aberration (mm)	2.7		2.7		2.7		2.7		2.7		2.7	
Electron Exposure ($e^-/Å^2$)	60		60		60		60		60		60	
Defocus range (μm)	-1.0 to -2.5		-1.0 to -2.5		-1.0 to -2.5		-1.0 to -2.5		-1.0 to -2.5		-1.0 to -2.5	
Pixel size (Å, Physical/Digital)	0.735		0.735		0.735		0.735		0.735		0.735	
Energy Filter Slit Width (eV)	10		10		10		10		10		10	
**Map Statistics and Post-Processing**												
Symmetry imposed	C1	C2	C1	C2	C1	C2	C1	C2	C1	C2	C1	C2
Map Resolution (Å)	3.01	2.78	2.91	2.7	2.55	2.37	2.52	2.37	2.72	2.56	2.75	2.61
Local resolution range for 75% of voxels	7.069	6.294	6.908	5.975	6.521	5.798	6.769	5.901	6.983	5.971	7.095	6.344
Local resolution range (model)	2.6 - 41.1	2.4 - 26.9	2.5 - 42.7	2.3 - 22.6	1.6 - 38.8	2.1 - 27.6	2.2 - 39.0	2.1 - 34.5	2.4 - 42.7	2.2 - 38.4	1.6 - 41.6	2.3 - 25.4
Map sharpening B factor ($Å^2$)	118.3	111.9	112.3	110.2	89.2	85.9	82.7	80.5	91.9	91.1	89.5	93.4
Map sharpening method	Resolve	Resolve	Resolve	Resolve	Resolve	Resolve	Resolve	Resolve	Resolve	Resolve	Resolve	Resolve
Q-Score	0.7	0.74	0.73	0.76	0.78	0.81	0.78	0.78	0.75	0.78	0.76	0.78
Movies	2847	2847	2384	2384	2475	2475	1538	1538	3925	3925	2220	2220
**Model Statistics and Validation**												
Model composition												
Non-hydrogen atoms	4543	4577	4547	4584	4604	4591	4853	4950	4587	4862	4580	4601
Protein	568	568	568	568	568	568	568	568	568	568	568	568
Nucleic acids	0	0	0	0	0	0	0	0	0	0	0	0
Ligand1	HEM: 4	HEM: 4	HEM: 4	HEM: 4	HEM: 4	HEM: 4	HEM: 4	HEM: 4	HEM: 4	HEM: 4	HEM: 4	HEM: 4
Waters	51	85	55	92	104	91	353	450	91	366	88	109
R.M.S deviations												
Length (Å)	0.004	0.039	0.033	0.005	0.047	0.022	0.003	0.003	0.024	0.003	0.024	0.039
Angles (°)	0.602	1.496	1.215	0.934	2.128	1.218	0.512	0.531	1.331	0.673	1.466	1.940
MolProbity score	1.24	1.27	1.30	1.36	1.30	1.18	1.36	1.48	1.23	1.27	1.18	1.19
MolProbity Clashscore	4.72	5.06	5.61	4.60	5.50	3.93	6.06	5.38	4.49	5.16	3.93	4.04
CaBLAM (% outliers)	0.91	0.93	0.73	0.72	0.75	0.73	0.54	0.72	0.36	0.72	0.55	0.74
Rotamer outliers (%)	0.66	0.44	0.44	1.32	0.44	0.88	1.10	1.76	0.44	0.00	0.44	0.00
Cis peptides (#, %)	0.0/0.0	0.0/0.0	0.0/0.0	0.0/0.0	0.0/0.0	0.0/0.0	0.0/0.0	0.0/0.0	0.0/0.0	0.0/0.0	0.0/0.0	0.0/0.0
Ramachandran Plot												
Favored	98.92	98.18	98.74	97.86	98.72	99.82	99.29	99.64	99.28	99.11	99.1	99.46
Allowed	1.08	1.82	1.26	2.14	1.28	0.18	0.71	0.36	0.72	0.89	0.9	0.54
Outliers	0.0	0.0	0.0	0.0	0.0	0.0	0.0	0.0	0.0	0.0	0.0	0.0

Table S1 CryoEM data collection and refinement statistics of No Oil, Oil, Very Low NaDT, Low NaDT, Partial Oxy, and DeOxy Hb.

	Oxidized MoFeP		Reduced MoFeP,60mM		Reduced MoFeP, 20mM		Reduced MoFeP, 5mM	
**Data Collection**								
Magnification	165 kx		165 kx		165 kx		165 kx	
Voltage (kV)	300		300		300		300	
Spherical Aberration (mm)	2.7		2.7		2.7		2.7	
Electron Exposure ($e^-/Å^2$)	60		60		60		60	
Defocus range (μm)	-0.75 to -2.5		-0.75 to -2.5		-0.75 to -2.5		-0.75 to -2.5	
Pixel size (Å, Physical/Digital)	0.735		0.735		0.735		0.735	
Energy Filter Slit Width (eV)	10		10		10		10	
**Map Statistics and Post-Processing**								
Symmetry imposed	C1	C2	C1	C2	C1	C2	C1	C2
Map Resolution (Å)	2.51	2.39	2.19	2.08	2.19	2.08	2.33	2.19
Local resolution range for 75% of voxels	6.69	6.034	6.097	5.443	6.273	5.658	6.305	5.681
Local resolution range (model)	2.1 - 39.0	1.6 - 38.1	1.6 - 35.1	1.8 - 35.0	1.9 - 35.2	1.7 - 35.2	2.0 - 39.0	1.6 - 35.1
Map sharpening B factor ($Å^2$)	65.2	68.3	50.2	51.3	51.0	51.8	63.1	64.2
Map sharpening method	Resolve	Resolve	Resolve	Resolve	Resolve	Resolve	Resolve	Resolve
Q-Score	0.8	0.82	0.83	0.85	0.85	0.85	0.82	0.82
Movies	2002	2002	2000	2000	2000	2000	2501	2501
**Model Statistics and Validation**								
Model composition								
Non-hydrogen atoms	17271	17365	17190	17043	17462	17763	17064	17206
Protein	1998	1998	1998	1998	1998	1998	1998	1998
Nucleic acids	0	0	0	0	0	0	0	0
Ligand1	ICS: 2	ICS: 2	ICS: 2	ICS: 2	ICS: 2	ICS: 2	ICS: 2	ICS: 2
Ligand2	CLF: 2	CLF: 2	CLF: 2	CLF: 2	CLF: 2	CLF: 2	CLF: 2	CLF: 2
Ligand3	HCA: 2	HCA: 2	HCA: 2	HCA: 2	HCA: 2	HCA: 2	HCA: 2	HCA: 2
Ligand4	FE: 2	FE: 2	FE: 2	FE: 2	FE: 2	FE: 2	FE: 2	FE: 2
Waters	1244	1338	1163	1016	1435	1736	1037	1179
R.M.S deviations								
Length (Å)	0.003	0.004	0.004	0.007	0.002	0.003	0.003	0.003
Angles (°)	0.509	0.586	0.703	0.971	0.497	0.560	0.637	0.633
MolProbity score	1.66	1.59	1.63	1.60	1.40	1.82	1.40	1.53
MolProbity Clashscore	6.85	9.63	8.18	4.80	6.41	14.96	6.09	6.98
CaBLAM (% outliers)	0.61	0.76	0.91	0.86	0.55	0.71	0.61	0.76
Rotamer outliers (%)	1.57	1.04	0.12	2.50	1.04	0.81	0.58	0.29
Cis peptides (#, %)	6.5/0.1	6.5/0.1	6.5/0.1	4.3/0.1	6.5/0.1	6.5/0.1	6.5/0.1	6.5/0.1
Ramachandran Plot								
Favored	97.29	97.69	96.93	97.79	97.84	97.24	97.69	97.24
Allowed	2.71	2.31	3.07	2.21	2.16	2.76	2.31	2.76
Outliers	0.0	0.0	0.0	0.0	0.0	0.0	0.0	0.0

Table S2 CryoEM data collection and refinement statistics of Oxidized and Reduced MoFeP

	Institution	Location	Website
**1.**	University of California, San Diego	San Diego, USA	https://cryoemfacility.ucsd.edu/instruments/index.html
**2.**	La Jolla Institute for Immunology	San Diego, USA	https://www.lji.org/research/researchservices/cryoem/
**3.**	Yale	New Haven, USA	https://medicine.yale.edu/ccmi/em/
**4.**	University of Michigan	Ann Arbor, USA	www.lsi.umich.edu/science/centers-technologies/cryo-electron-microscopy/microscopes-and-sample-prep/chameleon
**5.**	Baylor College of Medicine	Houston, USA	https://www.bcm.edu/research/atc-core-labs/cryoem-core/instrumentation-technology
**6.**	Florida State University	Tallahassee, USA	https://bsir.bio.fsu.edu/sptlabtech-chameleon
**7.**	University of California, San Francisco	San Francisco, USA	https://www.bcm.edu/research/atc-core-labs/cryoem-core/instrumentation-technology
**8.**	Massachusetts Institute of Technology	Cambridge, USA	https://nanousers.mit.edu/characterizenano/focus-facilities/automated-cryogenic-electron-microscopy/chameleon
**9.**	University of Washington	Seattle, USA	https://sites.uw.edu/jkoll/sample-page/
**10.**	New York University	New York, USA	https://med.nyu.edu/research/scientific-cores-shared-resources/cryo-electron-microscopy-laboratory/equipment
**11.**	electron Bio-Imaging Centre (eBIC)	Didcot, England	https://www.diamond.ac.uk/Instruments/Biological-Cryo-Imaging/eBIC/Instruments/Chameleon.html
**12.**	Centre for Integrative Biology / IGBMC	Toulouse, France	https://www.igbmc.fr/en/plateforms-and-services/platforms/integrated-structural-biology/translate-to-english-microscopie-electronique#tab-3899
**13.**	Cold Spring Harbor Laboratory (CSHL)	Cold Spring Harbor, USA	https://joshua-torlab.labsites.cshl.edu/
**14.**	National Cancer Institute	Frederick, USA	https://ccr.cancer.gov/center-for-structural-biology/cryo-em-facility/instrumentation
**15.**	Janelia Research Campus	Ashburn, USA	https://www.janelia.org/support-team/cryo-electron-microscopy/equipmentservice
**16.**	Shanghai Pasteur Institute, Chinese Academy of Sciences	Shanghai, China	http://english.siii.cas.cn/hxss/js/otc/
**17.**	Dubochet Center for Imaging (DCI) EPFL Swiss Federal Institute of Technology Lausanne	Lausanne, Switzerland	https://dci-lausanne.ch/resource/chameleon/
**18.**	SLAC National Accelerator Laboratory	Menlo Park, USA	https://cryoem-s2c2.slac.stanford.edu/project-requests
**19.**	New York Structural Biology Center	New York, USA	https://nccat.nysbc.org/instrumentation/
**20.**	NanoImaging Services	San Diego, USA	https://www.nanoimagingservices.com/platform/sample-preparation
**21.**	SciLifeLab	Solna, Sweden	https://www.scilifelab.se/units/cryo-em/

Table S3: Location and websites of institutions and national centers that possess a SPT Labtech chameleon.
